# Regulation of Pathologic Retinal Angiogenesis in Mice and Inhibition of VEGF-VEGFR2 Binding by Soluble Heparan Sulfate

**DOI:** 10.1371/journal.pone.0013493

**Published:** 2010-10-20

**Authors:** Koji M. Nishiguchi, Keiko Kataoka, Shu Kachi, Keiichi Komeima, Hiroko Terasaki

**Affiliations:** Department of Ophthalmology, Nagoya University Graduate School of Medicine, Nagoya, Japan; University of Giessen Lung Center, Germany

## Abstract

Development of the retinal vascular network is strictly confined within the neuronal retina, allowing the intraocular media to be optically transparent. However, in retinal ischemia, pro-angiogenic factors (including vascular endothelial growth factor-A, VEGF-A) induce aberrant guidance of retinal vessels into the vitreous. Here, we show that the soluble heparan sulfate level in murine intraocular fluid is high particularly during ocular development. When the eyes of young mice with retinal ischemia were treated with heparan sulfate-degrading enzyme, the subsequent aberrant angiogenesis was greatly enhanced compared to PBS-injected contralateral eyes; however, increased angiogenesis was completely antagonized by simultaneous injection of heparin. Intraocular injection of heparan sulfate or heparin alone in these eyes resulted in reduced neovascularization. In cell cultures, the porcine ocular fluid suppressed the dose-dependent proliferation of human umbilical vein endothelial cells (HUVECs) mediated by VEGF-A. Ocular fluid and heparin also inhibited the migration and tube formation by these cells. The binding of VEGF-A and HUVECs was reduced under a high concentration of heparin or ocular fluid compared to lower concentrations of heparin. In vitro assays demonstrated that the ocular fluid or soluble heparan sulfate or heparin inhibited the binding of VEGF-A and immobilized heparin or VEGF receptor 2 but not VEGF receptor 1. The recognition that the high concentration of soluble heparan sulfate in the ocular fluid allows it to serve as an endogenous inhibitor of aberrant retinal vascular growth provides a platform for modulating heparan sulfate/heparin levels to regulate angiogenesis.

## Introduction

The exposure of the retina to various insults such as chronic elevation of blood glucose or sudden exposure to high oxygen induces obliteration of pre-existing retinal vascular structures. It is often followed by the extension of retinal vessels from the border of the avascular retina into the vitreous cavity, which triggers a string of events that ultimately compromise vision. Pathologies that arise from these insults include diabetic retinopathy or retinopathy of prematurity, both of which are growing concerns worldwide because of the alteration in diet and lifestyle or the increased survival of premature infants due to advancements in neonatal medicine. While angiogenesis is strictly confined to the retina during development, little is known about why vascular regeneration favors aberrant extension into the vitreous in ischemic retinopathies.

Heparan sulfate (HS) proteoglycans are composed of a core protein and one or more sugar chains with specific patterns of linear polysaccharides called glycosaminoglycan (GAG) [1]. These proteoglycans are expressed ubiquitously on the surface of all cell types as transmembrane or membrane-anchored protein or within the extracellular matrix as secreted forms, critical for various physiological processes. Numerous sulfations present within the sugar chains provide a strong negative charge, allowing interaction with various heparin-binding proteins and their receptors [1]. Genetic ablation studies have revealed the specific role of each HS proteoglycan that depend, at least partly, on localization [2]. Aside from tissue specificity, compelling evidence indicates that membrane-associated HS promotes the interactions of various heparin-binding growth factors, including vascular endothelial growth factor-A (VEGF-A), to their receptors [3], [4]. However, the role of soluble HS in the interactions appears to be more dependent on the context, such as cell type, tissue, or its own concentration, and is bivalent at times, making interpretation difficult [5], [6]. The binding of soluble GAGs to the cell surface converting them to the membrane-associated form also contributes to the complexity [3]. For example, constitutive expression of the soluble HS proteogylcan, shed syndecan-1, decreased the proliferation of MCF-7 adenocarcinoma cells [6] while exposure of T47D ductal carcinoma cells to secreted syndecan-1 stimulated their proliferation [7]. Lower concentrations of soluble HS GAGs in culture media significantly promoted the binding of VEGF-A to cultured melanoma cells, while an opposite effect has been recognized at higher concentrations [8]. The function of a particular soluble HS proteoglycan *in vivo* is even harder to define; this is probably because the function of freely mobile HS proteoglycans is less dependent on the type of core protein, and the loss of a particular proteoglycan may be compensated by others.

The aqueous humor is a clear fluid that circulates the anterior compartment of the eye. It is actively produced in the posterior chamber by the non-pigmented ciliary epithelium and flows anteriorly through the pupil and reabsorbs through the collector channel in the angle. Estimation indicates about 1% turnover in aqueous volume per minute. The chemical content of the aqueous humor closely reflects the molecules found in the vitreous [9] but the concentrations are always lower in the former; this is likely mediated through gradient-driven anterior diffusion [9], [10]. Evidence suggests that soluble HS in the aqueous humor harbors an anti-angiogenic property, inhibiting the binding of pro-angiogenic factors (VEGF-A and basic fibroblast growth factor) to their cell surface receptors [11]. However, the physiological significance of soluble HS in the eye remains to be demonstrated.

In this study, the role of soluble HS/heparin GAGs on retinal angiogenesis was investigated. We found that the sufficiently high concentration of soluble HS in the ocular fluid serves as a potent endogenous inhibitor of aberrant growth of vessels from retinal surface into the vitreous. The results suggested that this occurs partly through inhibition of VEGF-A-receptor interaction and signaling.

## Results

### Heparan sulfate in the aqueous humor is increased during development

**Figure 1:**
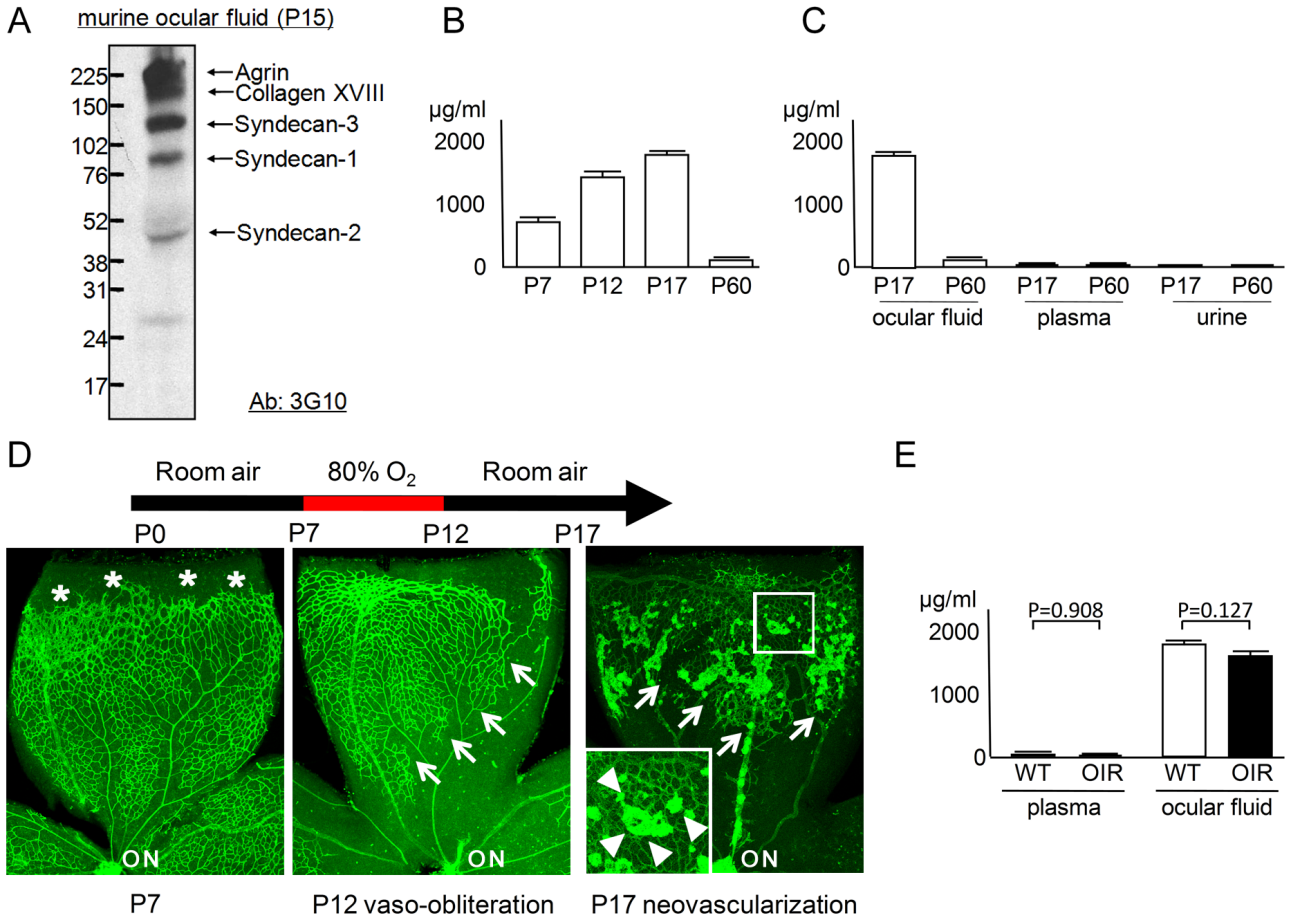
HS GAG is increased in ocular fluid. (A) Profile of HS proteoglycans in the ocular fluid. Western blotting of the ocular fluid from P15 mice revealed multiple bands that presumably correspond to agrin, collagen XVIII, syndecan-3, syndecan-1, and syndecan -2 [14], [15]. (B) Time-course profile of HS concentration in ocular fluid. HS level was increased in younger mice (P7, P12, and P17; N = 6 each) compared to older animals (P60; N = 5). (C) Comparison of HS concentration among body fluids. HS level was higher in ocular fluid compared to plasma or urine at P17 (N = 7) and P60 (N = 5), respectively. (D) GS staining of retinal quadrants in the OIR model. At the beginning of O_2_ exposure (P7), retinal vessels are extending toward the periphery (asterisk). The retinal vessels obliterate and regress (outlined by arrows) after exposure to 80% oxygen (P12). Returning the mice to room air results in outgrowth of extra-retinal NV at P17 (NV tufts are outlined by arrowheads in inset). Note that clumps of extra-retinal NV show stronger GS staining compared to intra-retinal vessels. (E) Comparison of HS concentration between samples from OIR and wild-type (WT) mice (N = 7 each). No difference was detected. ON: optic nerve. All statistical data are expressed as mean ± standard error of the mean (S.E.M.).

Newborn mice lack vascular structures in their retinas. Developing retinal vessels grow radially from the optic nerve toward the periphery along the vitreo-retinal interface, followed by the formation of a deeper vascular plexus over the first 2 weeks after birth. We first assessed the composition of HS proteoglycans secreted in the ocular fluid from a mouse aged Post-natal day 15 (P15). An antibody that detects the urinate residues on the HS core proteins created by the treatment of heparinase III resulting in a specific degradation of HS GAGs was used (Figure 1A) [12]. As with the previous report on vitreous samples from chickens [13], multiple HS core proteins were observed. Soluble agrin, collagen XVIII, or syndecans 1, 2, and 3 were deduced based on their molecular weights from previous reports [14], [15]; many of these proteoglycans have been reported to be expressed in the retina [13], [16]–[18]. Next, we studied the concentrations of HS GAGs in the ocular fluid collected from mice ranging in age from P7 to P60 using an enzyme-linked immunosorbent assay (ELISA) that specifically detects HS polysaccharides (Figure 1B). The level of HS was much higher in younger mice compared to older mice; the level was 15.8-fold higher at P17 compared to P60. The HS concentration in ocular fluid appeared to be uniquely high compared to other body fluids such as plasma (178-fold and 71-fold higher at P17 and P60, respectively) or urine (236-fold and 30-fold higher at P17 and P60, respectively) from the same mice (Figure 1C).

The exposure of mouse pups (P7) to 80% oxygen for 5 days results in an extensive obliteration and drop-out of the retinal vasculature around the optic nerve (Figure 1D). This is due partly to the rapid down-regulation of VEGF-A by retinal parenchyma [19] and oxidative insults [20]. Subsequently returning these mice to room air for another 5 days results in the up-regulation of VEGF-A from the non-perfused retina [21], [22] and the development of extra-retinal neovascularization (NV) and intra-retinal revascularization (oxygen-induced retinopathy, OIR; Figure 1D) [23]. With a hypothesis that the secreted HS level may be differently regulated during active pathologic angiogenesis, we measured HS concentrations in the ocular fluid and plasma in mice with OIR. However, no difference was detected between samples from an OIR model and control mice (Figure 1E).

### HS/heparin GAGs inhibit the development of extra-retinal NV in vivo

**Figure 2:**
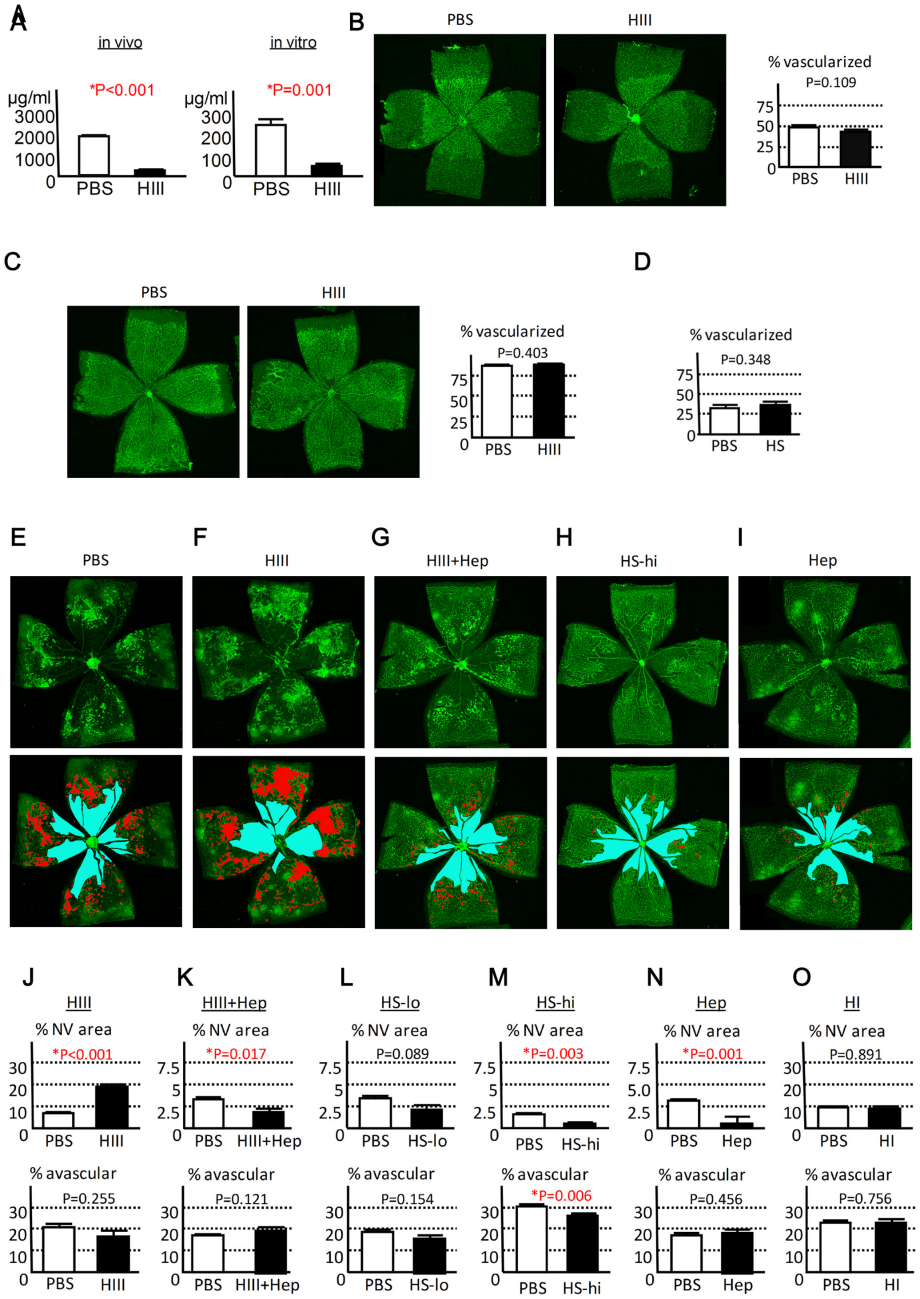
HS/heparin GAGs inhibit the development of NV in OIR. (A) Breakdown of HS GAGs by heparinase III (HIII). HIII reduced HS in the ocular fluid by 87.3% *in vivo* (left; N = 5) and 80.2% *in vitro* (right; N = 5). (B–C) Measurement of area covered with retinal vessels. Representative images of GS-stained retinas from P5 (B; N = 6) or P8 (C;N = 6) mice treated with PBS or HIII at P3 are shown and the proportion of vascularized area over the entire retina was quantified (%). Similarly, proportion of vascularized retina was measured in P5 mice treated with PBS or HS at P3 (D; N = 6). No difference was detected. (E–I) Representative appearance of GS staining of the retina treated with PBS (E), HIII (F), HIII and heparin (HIII+Hep; G), high-dose HS (HS-hi; H), or heparin (Hep; I) at P12 and analyzed at P17 (upper images). The same images were highlighted in red and blue for NV and avascular area, respectively. (lower images). (J–O) Quantification of NV area (upper panels) and avascular retina (lower panels) expressed as area relative to entire retina (%). The eyes were treated with HIII (N = 9), HIII+Hep (N = 8), low-dose HS (HS-lo; N = 8), HS-hi (N = 8), Hep (N = 7), and heparinase I (HI; N = 6). Note increased NV in the eyes treated with HIII (J), which was antagonized by co-injection of Hep (K). NV was decreased by intra-ocular injection of HS-hi (M) or Hep (N), while no difference was detected with HS-lo (L) or HI (O) administration. All statistical data are expressed as mean ± S.E.M.

In order to determine the role of HS GAGs in the ocular fluid, we applied heparinase III to non-specifically remove HS polysaccharides from the core protein. First, the efficacy of heparinase III as an endoglycosidase was determined. HS concentration was reduced by 87.3% when the enzyme was injected into the eyes *in vivo* and by 80.2% when the murine ocular fluid was incubated with heparinase III *in vitro* (Figure 2A). To elucidate the role of HS in physiological retino-vascular development in vivo, we injected heparinase III into the developing eyes at P3, when the vessels covered only a small proportion of the retina. Subsequent examination of the retina at P5 and P8 showed no difference in the area of vascularized tissue between the phosphate-buffered saline (PBS) - and heparinase III-treated eyes (Figure 2B and C). Intraocular injection of a high dose of exogenous HS GAGs purified from bovine kidney (resulting in ∼12,500 µg/ml in the eye) showed no detectable influence on the retinal vascular development (Figure 2D). Next, we evaluated the effect of HS degradation on pathologic angiogenesis in an OIR model. Heparinase III was injected into the eyes at P12 immediately after returning the mice to room air (at the end of 5 days' exposure to 80% oxygen). At P17, the degradation of HS by heparinase III dramatically increased the area of pathological NV by 2.9-fold compared to the PBS-treated contralateral eye (Figures 2F and 2J); this effect was completely abrogated by simultaneously injecting heparin, a heparinase III-insensitive GAG (Figures 2G and 2K). While heparin lacks the core protein, there is no qualitative difference between heparin and HS chains in their binding properties [24]. Therefore, the results strongly indicate that the inhibition of NV is mostly due to sugar chains and less dependent on the types of core protein. To extend this observation and see if HS or heparin GAG injection can negatively regulate pathologic angiogenesis, purified HS chains without the core protein or heparin alone were administered into the eyes with OIR at P12. As predicted, we observed a reduction of pathological NV by 64.2% with the administration of high-dose HS (∼12,500 µg/ml in the eye; Figures 2H and 2M), while the effect was variable and not evident at a lower dose (∼1,250 µg/ml in the eye; Figure 2L) at P17. The injection of high-dose heparin (∼12,500 µg/ml in the eye) in mice with OIR yielded a similar, but more potent (reduction of NV by 80.8%), and less variable effect (data not shown; Figures 2I and 2N). Endogenous heparin is produced exclusively by mast cells and basophils, but these cells are uncommon in the vitreous or retina. Accordingly, no effect was detected when the heparinase I endoglycosidase that targets heparin but not HS GAGs was injected (Figure 2O).

The influence of HS/heparin GAG modulation on the area of avascular retina (thus intra-retinal revascularization) was minimal (Figures 2J–O). Only high-dose HS injection showed a modest, but significant, effect on the area of avascular retina (reduction by 13.4%; Figure 2M). Administration of all other agents revealed no effect on this parameter.

### VEGF-A is paradoxically elevated in eyes injected with heparin

**Figure 3:**
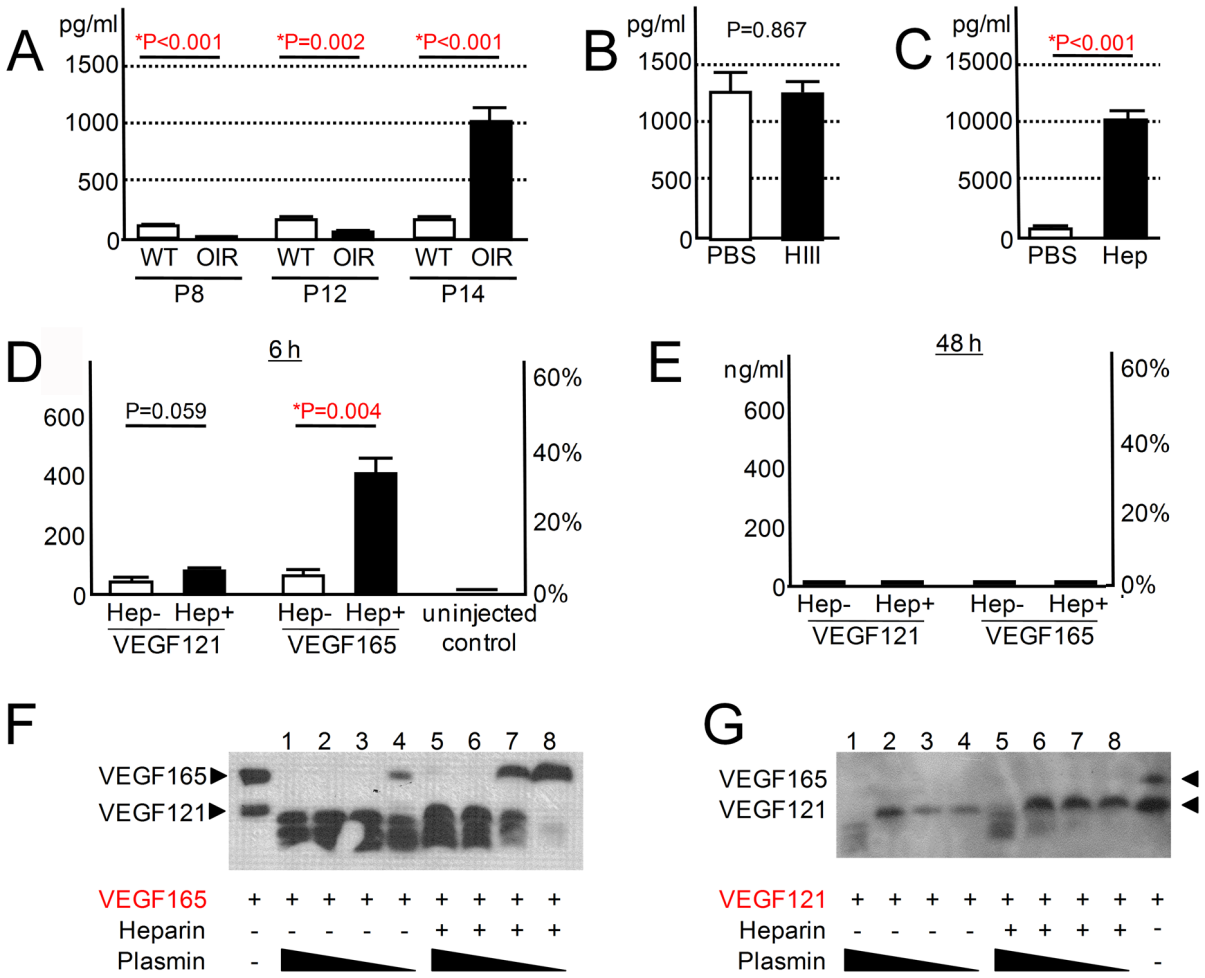
Soluble heparin-bound VEGF-A is inactivated but is also protected from proteolysis. (A) VEGF-A levels in the ocular fluid in OIR mice. VEGF-A concentration was determined at P8, P12, and P14 from wild-type control (WT) and OIR model, respectively (N = 6 each). (B,C) VEGF-A levels in the ocular fluid collected at P14 from OIR mice treated with heparinase III (HIII; N = 8) or heparin (Hep; N = 7) at P12. Intra-ocular heparin injection resulted in greatly increased VEGF-A levels (C) while levels were not affected by HIII treatment (B). (D,E) Kinetics of human VEGF-A in murine eyes at 6 and 48 hours after intraocular injection. Human isoform VEGF121 (5 ng/eye) was injected with heparin (20 µg/eye) in one eye and without in the other at P12. Ocular fluid was analyzed with human VEGF-specific ELISA at 6 and 48 hours later (N = 6 and N = 5, respectively). An identical experiment was conducted using human VEGF165 (5 ng/eye; N = 6 each). Only eyes co-injected with VEGF165 and heparin and analyzed at 6 hours post-injection showed increased human VEGF-A (D). The left and right vertical axes show concentration (ng/ml) and proportion (% relative to injected amount) of the remaining VEGF-A in the ocular fluid, respectively. Human VEGF-A was not detected in the uninjected control (N = 6). (F,G) The effect of heparin on *in vitro* degradation of VEGF-A by plasmin. VEGF165 (F), or VEGF121(G) was subjected to proteolysis by plasmin (400, 80, 16, and 3.2 ng for lanes 1 and 5, 2 and 6, 3 and 7, and 4 and 8, respectively). Undigested VEGF121 and VEGF165 were applied as references (arrowheads). All statistical data are expressed as mean ± S.E.M.

VEGF-A, a heparin/HS-binding glycoprotein, is the major pro-angiogenic growth factor that controls the development of extra-retinal NV in OIR [21], [22], [25]. The dominant role of this protein in the OIR model was confirmed by the intraocular injection of soluble VEGF receptor 1 (VEGFR1), a potent antagonist of VEGF-A, which resulted in 98.0% reduction in NV (Figure S1). Since HS/heparin GAGs are known to influence VEGF-A signaling, it is important to study the interactions of the related molecules to further understand the mechanistic basis for the anti-angiogenic role of soluble GAGs in the eye. First, we analyzed the level of VEGF-A in the ocular fluid in an OIR model and in wild-type controls. VEGF-A concentrations in the ocular fluid were reduced with exposure to 80% oxygen at P8 and P12 (Figure 3A). The subsequent introduction of the mice to room air rapidly increased VEGF-A level, resulting in 9.7-fold higher concentration compared to that of controls at P14. These observations are consistent with the reported alteration of VEGF-A production in the retina of OIR mice [19], [21], [22]. Next, we studied VEGF-A levels in the ocular fluid at P14, two days after the administration of heparinase III at P12 (immediately after returning the mice to room air), where elevated VEGF-A levels in OIR was not affected by heparinase III treatment, i.e., no difference was detected compared with PBS-treated eyes (Figure 3B). However, the ocular fluid from the heparin-injected eyes revealed a further 11.3-fold increase in VEGF-A concentration compared with the control OIR eyes with high VEGF-A levels (Figure 3C). Similarly, VEGF-A levels in the ocular fluid were increased by 2.6-fold (P<0.001) in the eyes injected with high-dose HS (4,085±232 pg/ml: mean ± S.E.M.) compared with PBS-treated OIR eyes (1,550±94 pg/ml). These paradoxical observations showing greatly increased VEGF-A in the heparin- or HS-treated eyes with reduced NV indicated that GAGs inhibit the function of VEGF-A as a vascular endothelial mitogen.

### VEGF165 binding to heparin inhibits its degradation

In order to explore the etiology of this paradoxical observation, we sought an explanation for the increased VEGF-A. First, retinal mVegf-A mRNA from P14 OIR mice injected with heparin or PBS at P12 was quantified. There was no obvious difference in the level of mVegf-A transcription between the eyes treated with heparin or PBS (P = 0.199; 1.2±0.7 or 4.5±2.5 fold-copies mCyclophilin A, repectively). This indicated that increased VEGF-A in the ocular fluid of heparin-treated eyes occurred by post-transcriptional mechanisms. VEGF-A isoforms, VEGF164 (VEGF165 in humans), and VEGF120 (VEGF121 in humans), are expressed during development of the retinal vasculature [22], [26]. VEGF164/165 is the major functional isoform that contains the heparin-binding domain. VEGF120/121 is the shorter isoform without this domain. Next, the kinetics of VEGF-A in murine eyes were examined by injecting human isoform VEGF121 or VEGF165 alone without heparin in one eye and with heparin in the other eye. Then the ocular fluid was collected 6 and 48 hours later and the remnant of injected VEGF-A was measured with an ELISA that detected only human isoforms. At 6 hours after the protein injection, human VEGF165 was 6.38-fold higher in the eye injected with heparin compared to the contralateral eye without (Figure 3D). Meanwhile, no difference in human VEGF121 level was detected between the eyes that received VEGF121 with or without heparin. At 48 hours after the injection, both isoforms were undetectable regardless of heparin co-administration (Figure 3E). The prolonged retention of VEGF165 by heparin but not VEGF121 is compatible with the direct binding of heparin to the heparin-binding domain of VEGF165 contributing to the phenomenon. Plasmin is a serine protease that is expressed in the eyes [27] and is known to cleave or degrade VEGF-A into smaller fragments [28], [29]. We tested whether heparin binding to VEGF-A can result in resistance to proteolysis by plasmin *in vitro*. Heparin inhibited the plasmin-mediated breakdown of the VEGF165 isoform (Figure 3F), but not VEGF121 (Figure 3G). Taken together, these results indicate that heparin-VEGF165 binding contributes to an increased intraocular VEGF-A concentration, at least partly through the inhibition of VEGF165 degradation by endogenous proteases such as plasmin.

### High level of soluble heparin inhibits proliferation, migration, and tube formation of vascular endothelial cells

**Figure 4:**
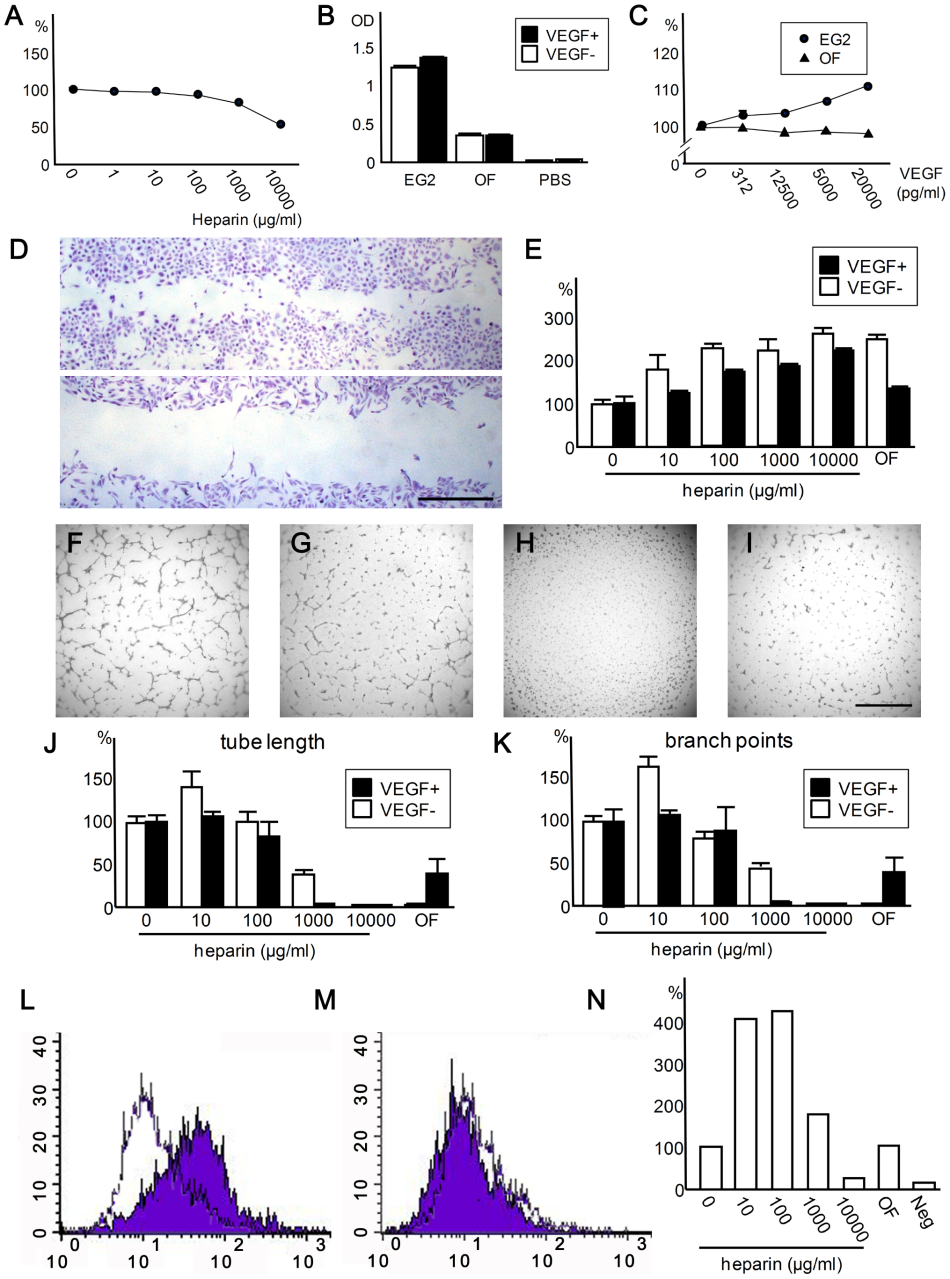
Heparin and ocular fluid inhibit proliferation of HUVECs. (A) Effect of heparin on the viability of HUVECs. Various concentrations of heparin were added to the cells in EG2 media supplemented with VEGF-A (10 ng/ml). Note viability of HUVECs is suppressed by soluble heparin in a dose-dependent manner. The experiment was done in quadruplicate and repeated. (B) Comparison of viability among HUVECs cultured with different medium. Note that viability of HUVECs is increased with addition of VEGF-A (10 ng/ml) when cultured with EG2 media but not with PBS or ocular fluid (N = 6 for each condition). Viability was low when cultured with PBS. The experiment was repeated. (C) Viability of HUVECs cultured in EG2 media or ocular fluid (OF) with increasing concentrations of VEGF-A. Note that a positive dose-response relationship was observed when cultured in EG2 media, while HUVECs were unresponsive to VEGF-A in the ocular fluid. OD measurements relative to a condition without heparin (A) or VEGF-A (C) are shown in percentages (%). Experiments were done in quadruplicate and repeated. (D,E) Effect of heparin and ocular fluid on scratch wound induced migration of HUVECs. Representative images of HUVECs 24 hrs after creating scratch wound (D). Groove was larger when cells were incubated with ocular fluid (lower panel) compared with PBS (upper panel). Dose-dependent inhibition of cell-migration by heparin was observed with or without co-administration of VEGF-A (E). The assay was done in triplicate and repeated. (F–K) Effects of heparin and ocular fluid on tube formation of HUVECs. Representative images of HUVECs cultured in media with 0 (F), 1,000 (G), and 10,000 (H)µg/ml heparin or in ocular fluid (I), all of which contained VEGF-A. Note that only 10,000 µg/ml heparin completely suppressed tube formation. The degree of tube formation was quantified by measuring tube length (J) and counting branch points (K). At the lowest dose tested, heparin promoted the tube formation while an opposite effect was observed at higher doses in culture media. Note that ocular fluid suppressed angiogenesis incompletely and completely with and without VEGF-A, respectively. The assays were conducted in triplicate and repeated. (L–N) Effects of heparin and ocular fluid on the binding of VEGF-A and HUVECs. Representative images of FACS analyses of fluorescein-labeled VEGF-A and HUVECs showing increased and decreased binding at 10 (M) and 10,000 (N) µg/ml heparin (purple) compared to condition without heparin (white), respectively. Analysis showed that presence of lower concentrations (10 and 100 µg/ml) of heparin promoted the bindings while showing inhibitory effect at the highest dose (10,000 µg/ml; N). Each data point is an average of two measurements. The experiment was repeated. Data presented in (A–C,) (E), (J), and (K) are expressed as mean ± S.E.M. Scale bars indicate 1 mm.

Next, we assessed the effect of ocular fluid and soluble heparin on the VEGF-A-mediated viability of human umbilical vein endothelial cells (HUVECs). Culture studies are suitable for reproducing the unique ocular environment in which acellular vitreous/ocular fluid overlays the plane of retinal vascular network. HUVECs were incubated with increasing concentrations of heparin in a standard endothelial culture media (EG2) supplemented with VEGF-A. The viability of HUVECs showed an inverse dose response to heparin, which revealed 31.2% reduction in optical density (OD) measurements at a heparin level (1,000 µg/ml) relevant to the HS concentrations in the ocular fluid from OIR models (Figure 4A).

Next, the viability of HUVECs cultured in PBS, EG2, or porcine ocular fluid with or without VEGF-A (10 ng/ml) was assessed (Figure 4B). While few HUVECs survived in PBS alone, ocular fluid maintained the viability of the cells (albeit to a lesser degree when compared to EG2). Only HUVECs cultured in EG2 were sensitive to VEGF-A stimulation. A dose-response curve showed a positive correlation between VEGF-A level and the viability of HUVECs in EG2 media (Figure 4C). Conversely, HUVECs were unresponsive to VEGF-A when incubated in ocular fluid. These results are compatible with the inhibition of VEGF-A signaling by soluble GAG as one of the mechanisms for the anti-angiogenic effects of ocular fluid.

In order to further assess the effect of heparin and ocular fluid on the functional aspects of vascular endothelial cells, we first evaluated the migration of HUVECs induced by creating an artificial groove in the middle of the well. Heparin suppressed the migration of the cells into the groove in a dose dependent manner in media with or without VEGF-A (1,000 pg/ml; Figure 4D and 4E). However the inhibitory effect was less pronounced in the former. Ocular fluid also suppressed the migration of HUVECs. Next, we assessed the in vitro tube formation by HUVECs (Figure 4F–K). While heparin increased the length of tubular network and number of branch points, thus enhanced the tube formation, at lower concentration (10 µg/ml), the presence of higher concentration of heparin (1,000 and 10,000 µg/ml) or ocular fluid inhibited the formation (Figure 4J and 4K). We found similar but less inhibitory effects when VEGF-A (1,000 pg/ml) was added to the medium. Notably, no tube structure was formed under highest heparin concentration and ocular fluid when no VEGF-A was added. Meanwhile, trace evidence of tube formation was observed in ocular fluid under the presence of VEGF-A (Figure 4F–K). The results of cell-based assays conformed well to the results of in vivo injection studies showing suppression of angiogenesis by endogenous HS in the ocular fluid and further reduction achieved by exogenously administered GAGs, but only at higher concentrations.

### High level of soluble heparin inhibits binding of VEGF-A and HUVECs

The results of *in vivo* and cell-based experiments suggest that administration of soluble HS/heparin inhibits angiogenesis, in part, by blocking the functional activity of VEGF-A. In order to investigate the mechanistic basis for this observation, we assessed the interaction of VEGF-A and HUVECs (Figure 4L–N). We found that the lower concentrations of heparin (10 and 100 µg/ml) enhanced the binding of VEGF-A and HUVECs by ∼ 4-fold compared to the condition without heparin; this effect decreased dramatically with increasing concentration of heparin. At the highest concentration studied (10,000 µg/ml heparin), VEGF-A bound to HUVECs were decreased by 72.9% compared to the control with no heparin. Meanwhile, in the presence of ocular fluid, VEGF-A bound to HUVECs with similar efficiency as the control condition.

### Soluble heparin inhibits the binding of VEGF165 to surface heparin

**Figure 5:**
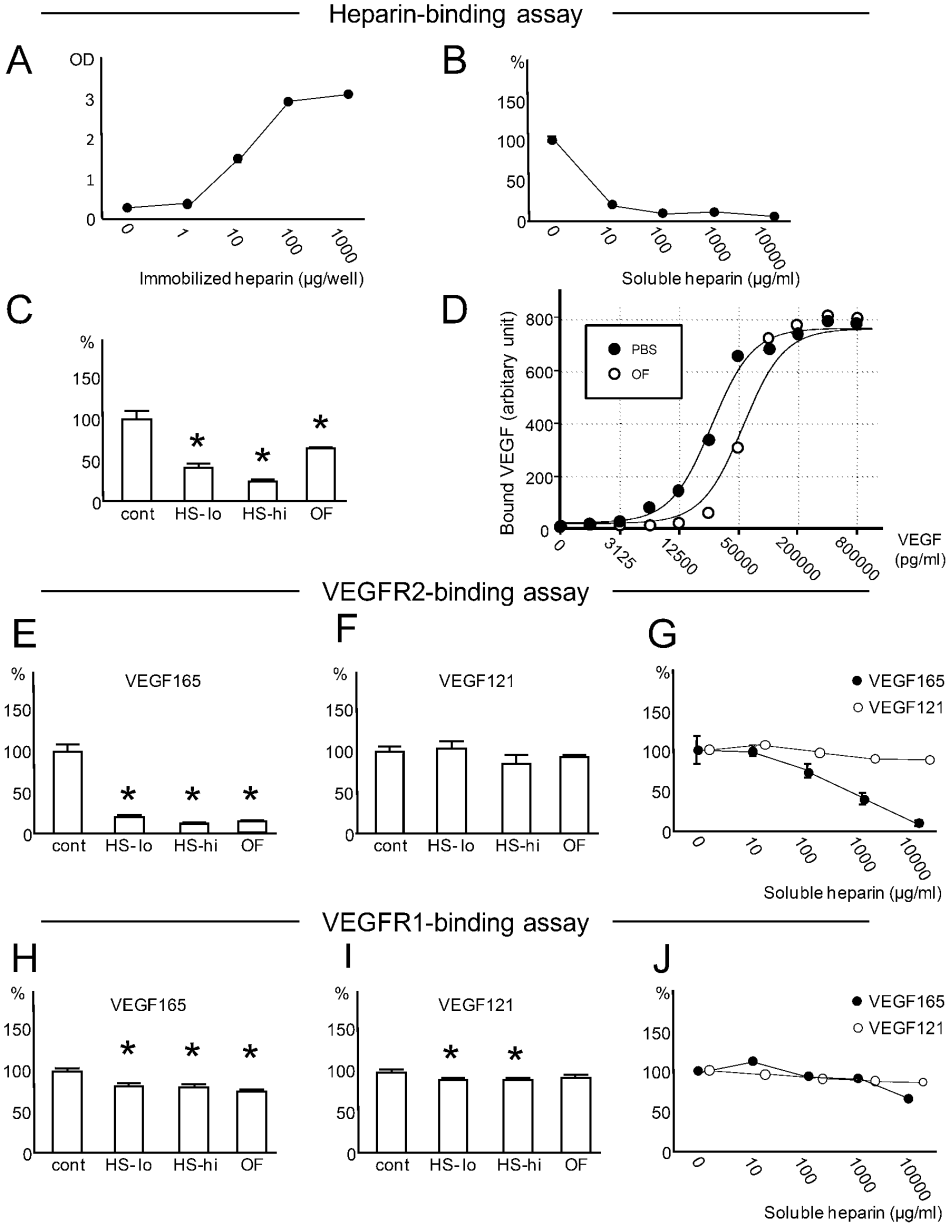
Soluble HS/heparin GAGs inhibit the binding of VEGF165 to immobilized heparin and VEGFR2. (A) Binding of VEGF-A and increasing amounts of immobilized heparin (horizontal axis). (B) Influence of soluble heparin on binding of VEGF-A and immobilized heparin. VEGF-A was applied with an increasing level of soluble heparin (horizontal axis) to a fixed amount of surface-coated heparin. (C) Binding of immobilized heparin and VEGF-A applied with soluble HS GAGs or ocular fluid (OF). Note that low (HS-lo;100 µg/ml) or high (HS-hi; 1000 µg/ml) concentrations of HS or OF reduced the binding of VEGF-A and immobilized heparin. (D) An increasing level of soluble VEGF-A and its association with immobilized heparin. VEGF-A (horizontal axis) was dissolved in PBS or OF. Regression analyses of VEGF-A in OF were consistent with rightward shift of the curve plotted with VEGF-A in PBS. (E–J) Influence of OF, soluble HS, or soluble heparin on the binding of VEGF165 or VEGF121 and immobilized VEGR2 (E–G) or VEGFR1 (H–J), respectively. Binding of VEGF165 and immobilized VEGFR2 was significantly inhibited with HS-lo, HS-hi, OF (E) and heparin (G). Meanwhile, binding of VEGF165 or VEGF121 and VEGFR1 was only modestly inhibited by HS/heparin GAGs or OF (H–J). Values relative to controls (%) are shown in (B), (C), and (E–J). Representative data from three experiments analyzed with an online software (http://zunzun.com) is presented in (D). For all other data, experiments (N = 6 for each data) were repeated and representative data are expressed as mean ± S.E.M. *P<0.050.

In order to further delineate the roles of surface-associated versus soluble GAGs on VEGF-A at a molecular level, wells were covalently crosslinked with heparin and the kinetics of VEGF-A-heparin binding under the presence or absence of soluble heparin were investigated with *in vitro* binding assays. When a constant amount of soluble VEGF-A was applied alone, surface-bound VEGF-A was positively correlated with the amount of heparin coating on the well bottom (Figure 5A). This finding corroborates the fundamental role of surface GAGs to provide docking sites on the cell surface for heparin-binding growth factors (including VEGF-A). Next, we studied the effect of soluble heparin on the binding of VEGF-A and immobilized heparin. An increasing concentration of heparin and a constant level of VEGF-A were added to a fixed amount of well-coated heparin. Soluble heparin suppressed the binding of VEGF-A and surface heparin dose dependently (Figure 5B). The binding was inhibited by 87.9% with 1, 000 µg/ml of soluble heparin. Parallel experiments showed that the bindings of VEGF-A and surface-heparin were inhibited also by the presence of soluble HS or porcine ocular fluid (Figure 5C).

To further characterize the nature of ocular fluid as an inhibitor of VEGF-heparin binding, a dose-binding curve was plotted by applying increasing concentrations of VEGF165 dissolved in PBS or porcine ocular fluid to heparin-coated wells (Figure 5D). Consistent with earlier experiments, the binding of VEGF-A and surface heparin was suppressed by ocular fluid. The result of regression analyses was compatible with the rightward shift of the binding curve with the presence of the ocular fluid (P<0.001; R^2^ = 0.976). This strongly indicates that the ocular fluid interferes with the association of surface heparin and VEGF-A by competing for the same heparin-binding domain of VEGF-A.

### Soluble HS/heparin GAGs inhibit the binding of VEGF165 and VEGF receptor 2 (VEGFR2)

VEGFR1 and VEGFR2 are well characterized receptors for VEGF-A. VEGFR2 is the key receptor for endothelial proliferation and migration [30]. VEGFR1, with more variable effects on endothelial cells, serves as a decoy receptor to regulate the activity of VEGF-A [30]. Both VEGFR1 and VEGFR2 are heparin-binding proteins [31], [32]. In order to define the role of soluble HS/heparin on the binding of VEGF-A and its surface receptors, VEGFRs were crosslinked to the bottom of wells and soluble HS GAGs or porcine ocular fluid were added with a fixed amount of VEGF-A. Soluble heparan sulfate or the ocular fluid inhibited the binding of VEGF165 and VEGFR2 (Figure 5E); in contrast, VEGF121-VEGFR2 association was not influenced (Figure 5F). Next, a constant level of VEGF-A (VEGF165 or VEGF121) and an increasing amount of heparin were applied to VEGFR2-coated wells. Soluble heparin inhibited the binding of VEGF165 and VEGFR2 in a dose-dependent manner, while showing minimal effect on VEGF121-VEGFR2 interaction (Figure 5G). At the heparin concentration of 1,000 µg/ml, the binding of VEGF165 or VEGF121 and VEGFR2 was reduced by 59.8% or 9.4%, respectively.

Parallel experiments to assess the effect of soluble GAGs on VEGF-A-VEGFR1 interaction revealed only modest or no suppression by HS, ocular fluid, or heparin, in every condition tested (Figures 5H–J).

Taken together, our results suggest that soluble HS/heparin GAGs or ocular fluid can directly prevent the binding of VEGF165 to VEGFR2, but not VEGF121. Meanwhile, the inhibition of VEGF165-VEGFR1 interactions was less evident.

## Discussion

In this study, we analyzed the role of soluble HS GAGs in an intraocular environment in which the plane of vascular network is embedded within the surface of the retinal parenchyma, but is also adjacent to the overlying ocular fluid that contained a high level of HS.

### Roles of endogenous HS as a suppressor of extra-retinal angiogenesis

Earlier studies showed presence of HS in the retina including collagen XIII, agrin, glypicans, and syndecans. [18] For example, the expression of syndecan 3 was increased during ocular development detected by immunohistochemistry. [16], [17] Ganglion cells, amacrine cells, and horizontal cells were shown to be the main source of syndecan 3, [16] which was also found in the ocular fluid. In the current study, concentration of soluble HS GAGs in the ocular fluid presumably derived from intraocular structures including the retinal parenchyma was quantified. We found extremely high levels of HS in the ocular fluid during ocular development compared to plasma or urine from the same group of mice, ocular fluid from adult mice, or reported HS levels in bovine aqueous humor [11]. This temporal expression profile simulated that reported for syndecan 3. [16] In this unique intraocular setting, the high concentrations of endogenous soluble HS GAGs appear to act as suppressors of pathologic angiogenesis, particularly under excessive amount of VEGF-A driven by retinal ischemia. In vitro studies suggested the ocular fluid and relevant levels of soluble GAGs could compete for VEGF-A with its surface counterpart that function as co-receptors for VEGFR2 or by directly interfering with ligand-receptor interactions. Since VEGFR2 is responsible for vascular endothelial proliferation and migration, suppression of VEGF-A-VEGFR2 interaction by soluble HS in the ocular fluid renders protection of the intra-vitreal environment from aberrant invasion of retinal vessels. The binding of VEGF-A and VEGFR1 was minimally affected by soluble GAGs suggesting that the binding affinity of this ligand-receptor is probably much higher than that of VEGF-A and GAGs. The relative increase in signaling of VEGFR1 over VEGFR2 may provide an additional advantage by defending retinal vessels from vaso-obliteration [33]. Meanwhile, modulation of the HS level in the vitreous cavity posed minimal influence on intra-retinal vascular growth during physiological development and vascular regrowth in OIR. These findings suggest that the anti-angiogenic property of soluble HS in ocular fluid preferentially inhibit extra-retinal extension of vessels, which corroborates with the unique distribution of ocular fluid. However, since significant amount of soluble HS (∼12.7%) still remain after the application of HS-degrading enzymes in the current experimental setting, further investigation is required to draw a conclusion on the role of soluble HS in intra-retinal vascularization.

### Potential of soluble GAGs as inhibitors of angiogenesis

Intraocular injection of soluble GAGs resulted in a reduced pathologic NV formation in OIR, showing a treatment effect for the ischemia-induced retinal vaso-proliferative disease in vivo. However, extremely high levels of GAGs (∼12,500 µg/ml in the eye) were required to achieve a clear therapeutic benefit. This was in agreement with the results of cell-based and in vitro binding assays showing that only higher doses of soluble heparin suppressed the angiogenic properties of HUVECs and interactions of VEGF-A and related molecules more efficiently than the ocular fluid. Meanwhile, the effects of lower concentrations of heparin was variable rendering both increased and decreased activity of HUVECs or interaction of VEGF-A that appeared to be dependent largely on the experimental conditions. These observations may explain somewhat inconsistent roles of heparin on angiogenesis and VEGF-A interactions proposed by different groups using lower doses of heparin [4], [34]–[36].

Meanwhile, a discrepancy between the level of HS in the ocular fluid and its ability to inhibit angiogenesis and molecular interactions of VEGF-A was noted. For example, inhibitory effect of ocular fluid (63.2 µg/ml of HS) on VEGF-A-VEGFR2 binding (∼85.7% inhibition) was comparable to 1,000 µg/ml of HS (∼88.1% inhibition) from bovine kidney in vitro. On the other hand, in vitro experiments also demonstrated that anti-VEGF-A feature of ocular fluid is due in part to competition with cell surface GAGs for VEGF-A binding, which is most likely by soluble GAGs found in the ocular fluid. As the structure/function of HS sugar chains on the same core proteins differs significantly between different organs or tissues [4], [37], injected GAGs from extra-ocular source used in this study may not have been as effective as those from the ocular fluid. Alternatively, an another possibility is that ocular fluid contains heparin-like substance that are different from HS or a factor that cooperate with HS to inhibit angiogenesis and function and molecular interaction of VEGF-A.

### Molecular mechanisms of functional inhibition of VEGF-A by GAGs

While addition of high levels of soluble GAGs was shown to exert anti-angiogenic effect and suppression of VEGF-A function, soluble GAGs can bind to cell surface and become membrane-associated. Therefore, the exact relationship between the spatial distribution of GAGs and VEGF-A with regard to the vitreo-retinal interface during the functional inhibition of VEGF-A by GAGs remains unclear. Similarly, mechanisms underlying heparinase III-mediated enhancement of pathological NV are unknown. Enzyme treatment likely results in the break-down of both soluble and surface HS. However, several lines of evidence suggested that presence of soluble GAGs in the fluid are important in the inhibition of pathological angiogenesis in OIR. First, the administration of soluble GAGs resulted in their binding to and retention of VEGF-A within the murine ocular fluid. Second, in vitro binding assay showed that administration of soluble GAGs inhibited the interaction of surface GAGs and VEGF-A. While spatial distribution of the GAGs with respect to well-surface was not investigated, it is likely that inhibition of the interaction occurred at the liquid phase because the amount of surface-bound VEGF was positively linked to that of surface-associated heparin in this experimental condition. Third, the injected GAGs targeted the pathological retinal vessels extending into the vitreous with high specificity while showing minimal effect on physiological vasculatures on the surface retina in vivo.

Both HS and heparin sugar chains share similar basic structures and interact with the same heparin-binding proteins, often showing similar effects. [1], [24] This is consistent with the observation that degradation of endogenous HS resulting in an increased pathological NV in OIR could be reversed by co-administration of heparin in vivo. The GAGs also shared similar effect in their basic quality toward the interaction of VEGF and surface GAGs or receptors. However, there are differences between these GAGs that could affect their affinities to a particular protein, thus efficiency of physiological effect. For example, the degree of sulfation is higher in heparin compared to HS resulting in greater negative charge density. [1] As ionic interaction plays a central role in the interaction of GAGs and heparin-binding proteins, heparin has higher affinity to these molecules compared to HS GAGs in general. We have confirmed that the variable decrease in N- and O-sulfation of GAGs does reduce their binding to VEGF-A in vitro (data not shown). This could be one explanation for the observation that HS was less efficient in blocking the function of VEGF-A and abnormal retinal angiogenesis compared to heparin.

### Disparities between VEGF-A level in the ocular fluid and angiogenic activity

An increased VEGF-A level in the ocular fluid (11.3-fold increase) in the heparin-injected eyes with reduced NV (reduction by 80.8%) was observed in OIR models, which was attributable in part to the resistance of heparin-bound VEGF-A to proteolysis, but not increased transcription of mVegf. Possibly due to a similar mechanism, an increase in VEGF-A level in cell culture media by the addition of soluble heparin has been reported previously [28]. Meanwhile, degradation of endogenous HS by heparinase III resulting in a 2.90-fold increase in ischemia-induced extra-retinal NV formation induced no alteration of VEGF-A level. Together with the results of cell-based and in vitro assays, it appears that the soluble GAGs in the vitreous bind to VEGF-A and raise its level while simultaneously neutralizing its function as an angiogenic factor. Thus, the disparities between VEGF-A concentrations and angiogenic activities are consistent with the presence of both functionally active and inactive form of VEGF-A unbound and bound with soluble GAGs, respectively. This indicates that the VEGF-A level in the aqueous humor or vitreous fluid per se may not accurately reflect the bioactivity of the VEGF-A signaling in the eye. These findings provide direct implications on the rapidly expanding use of anti-VEGF agents to treat vaso-proliferative diseases rationalized by elevated levels of intraocular VEGF-A.

In conclusion, soluble HS GAGs in the ocular fluid serve as endogenous inhibitors of aberrant retinal angiogenesis, partly mediated by the inhibition of interaction between VEGF-A and its receptors or HS GAGs on the cell surface. Our study provides a platform for modulating soluble HS/heparin levels to promote or inhibit angiogenesis in human disease.

## Materials and Methods

### Animals

All experimental procedures adhered to the ARVO Statement for the Use of Animals in Ophthalmic and Vision Research and the guidelines for the Use of Animals at Nagoya University School of Medicine. The research protocol on the use of animals has been approved by the Animal Facility Committee, Nagoya University School of Medicine (Approval # 22139). C57BL/6J mice used were kept under a 12-hour light–dark cycle. For the generation of OIR, P7 mice were first exposed to 80% oxygen (Proox 110; Bio Spherix Ltd., Lacona, NY, USA) for 5 days. Mice (P12) were then returned to room air for another 2 or 5 days until analyzed. OIR is a model for retinopathy of prematurity, which develops in prematurely born infants exposed to high oxygen, essential for life support. An agent was injected (0.5 µl) into one eye and PBS (Gibco Laboratories, Grand Island, NY, USA) into the other (at P3 or P12) after mice were anesthetized with intraperitoneal injections of tribromoethanol (Sigma-Aldrich Corp., St. Louis, MO, USA) at a dose of 250 mg/kg. The amounts of agents administered per eye were 0.005 U for heparinase III, 5 µg for low-dose HS, 50 µg for high-dose HS, 50 µg for heparin, and 0.005 U for heparinase I, and 0.005 U and 50 µg for co-injection of heparinase III and heparin, respectively, unless indicated otherwise. To minimize the variability of the experimental results, a single litter of mice (5–9 mice) was analyzed for each experiment.

### Materials

All VEGF-A used in this study were VEGF165 isoform unless designated otherwise. The recombinant VEGF165 and VEGF121 were purchased from PeproTech Inc., (Rocky Hill, NJ, USA) for the degradation and binding assays. VEGF receptor 1 (VEGFR1) and VEGFR2 were from R&D Systems Inc. (Minneapolis, MN, USA). HS GAGs purified from bovine kidney were from Seikagaku Corp. (Tokyo, Japan) and unfractionated heparin from porcine intestine, heparinase III, heparinase I, and chondoroitinase ABC were from Sigma-Aldrich Corp.. The murine plasmin was from Pierce Biotechnology (Rockford, IL, USA) The ocular fluid was collected and pooled from multiple porcine eyes (8-month-old animals) within 3 hours after the pigs were killed at a local slaughterhouse. After the ocular fluid was sterile filtered, the concentration of pooled HS was determined (63.2 µg/ml) and stored in −80°C. Porcine ocular fluid was later used for cell-based and binding assays.

### Western blotting and enzyme-linked immunosorbent assay (ELISA) of samples from body fluids

The optic nerve was removed together with a small island of sclera, choroid, and retinal pigment epithelium from the enucleated murine eye. A pipette tip was quickly inserted into the eye through the exposed retina, where the ocular fluid (2–5 µL per eye; a presumed mixture of vitreous fluid and aqueous humor) was aspirated. The ocular fluid were centrifuged at 13,000 g for 5 minutes and the supernatant was subjected to analysis.

The mouse urine was aspirated by applying a pipette in front of the genitals before sacrificing the animal, and peripheral blood was collected from the orbit after enucleation. The plasma was isolated from whole blood by centrifugation of the sample at 13,000 g for 5 minutes. These samples were stored in −80°C until analyzed.

The level of VEGF-A in the body fluids were measured with ELISA kits purchased from R&D Systems Inc and HS concentrations in the body fluids with those from Seikagaku Corp. according to the manufactuer's protocols. For western blotting of ocular fluid or aqueous humor, the samples were additionally treated with heparinase III (1 U/ml) and chondoroitinase ABC (2 U/ml) in 5 mM calcium acetate and 50 mM sodium acetate at 37°C overnight. After the samples were mixed with equal volume of Laemmli buffer (Biorad Laboratories Inc., Hercules, CA, USA), they were boiled, subjected to sodium dodecyl sulfate polyacrylamide gel electrophoresis using 4–20% acrylamide gradient gel (Biorad Laboratories Inc.) with a reducing condition. The protein was transferred to polyvinylidene fluoride membrane using the i-blot system (Invitrogen, Carlsbad, CA, USA). An antibody (3G10; 1∶500; Seikagaku Corp.) that reacts with unsaturated urinate/HS “stubs” generated by heparinase III treatment was used to detect HS proteoglycans (12).

### HS degradation assay

Mice were injected with heparinase III (0.005 U per eye) in one eye and PBS in the other at P12. After the ocular fluid was collected at P14, HS concentration was determined with ELISA (in vivo assay).

The ocular fluid (1.5 µl) collected from P14 mice was mixed with heparinase III (0.0025 U) or an equal volume of PBS, which was incubated overnight at 37°C in 5 mM calcium acetate and 50 mM sodium acetate (final volume was 10 µl). The remaining HS GAGs were measured with ELISA (in vitro assay).

### Flatmount analyses

The flatmount analyses were performed as previously described, with minor modifications (49). Briefly, the eyes were fixed in 4% paraformaldehyde for 2 hours and were stained overnight with FITC-labeled Griffonia simplicifolia lectin (GS; a marker for vessels and microglia/macrophages; 1∶100; Sigma-Aldrich Corp.). A single image that captured the entire retinal flatmount was obtained with a confocal microscope (Nikon, Tokyo, Japan) for each specimen. The areas of avascular retina and extra-retinal NV relative to the entire retinal were determined using Photoshop CS (Adobe Systems, San Jose, CA, USA).

### Real-time PCR

Total RNA was extracted with Trizol (Invitrogen) after tissue homogenization according to the manufacturer's instruction. Purified RNA (1 µg) was treated with DNase I (Invitrogen) and cDNA was synthesized with reverse transcriptase (Invitrogen) and 5 µmol/L random hexamer. Real-time PCR was performed and analyzed using the SYBR Green I format on the Light Cycler rapid thermal cycler system (ST300, Roche Diagnostics Ltd, Lewes, UK) according to the manufacturer's instructions. Reactions were performed using the SYBR Green reaction mix (Qiagen, Valencia, CA, USA) with 0.5 mmol/l primers. Murine Cyclophilin A RNA was used as a standard for normalization. The sequences of the PCR primer pairs were mVegf, 5′ -TTA CTG CTG TAC CTC CAC C-3′ (forward) and 5′ -ACA GGA CGG CTT GAA GAT G-' (reverse) and mCyclophilin A, 5′-CAG ACG CCA CTG TCG CTT T-3′ (forward) and 5′ -TGT CTT TGG AAC TTT GTC TGC AA-3′ (reverse). Samples that do not meet desirable PCR quality and specificity verified by melting-curve dissociation analysis were excluded from the analyses. For quantification, a standard curve was generated from a cDNA template for each gene. Titrations were performed to ensure that PCR were carried out in the linear range of amplification. Relative transcript levels of each gene were calculated using the second derivative maximum values from the linear regression of cycle number versus log concentration of the amplified gene. The amount of mVegf RNA was expressed as fold change relative to the number of copies measured for mCyclophilin A RNA (mean ± S.E.M.) from the same retina treated with heparin (N = 6) or PBS (N = 5). Comparable results were obtained in a replicate experiment using a different set of mice.

### In vitro VEGF-A degradation assay

Various amounts of murine plasmin (3.2, 16, 80, or 400 ng) were added to recombinant VEGF165 (20 ng) or VEGF121 (20 ng) with or without heparin (100 µg) in a reaction buffer (100 mM NaCl, 50 mM Tris-HCl, 10 mM MgCl2, 1 mM Dithiothreitol). The prepared samples were incubated at 37°C overnight and were subjected to western blotting using anti-VEGF-A antibody (sc-507; 1∶500; Santa Cruz Biotechnology, Inc., Santa Cruz, CA, USA), as described above.

### HUVEC proliferation assay

HUVECs purchased from Kurabo Biomedical (Osaka, Japan) were used in passages 3 to 5. Cells in EG2 media that contained epidermal growth factor (10 ng/ml), hydrocortisone (1 µg/ml), basic fibroblast growth factor (3 ng/ml), and heat-inactivated fetal bovine serum (1%) (all from Kurabo Biomedical), PBS, or porcine ocular fluid were seeded into 96-well plates (5,000 cells/100 µl/well) and incubated at 37°C. After 2 hours, heparin, VEGF-A (Kurabo Biomedical), or PBS was added to the medium. Cells were incubated for total of 48 hours before tetrazolium-based dye (WST-1; Promega Corp., Fitchburg, WI, USA) was applied. The amount of fromazan dye produced (reflecting the number of the viable cells) was measured with a plate reader (Bio-Rad Laboratories) 4 hours later.

### HUVEC scratch assay

After HUVECs were harvested by trypsin-EDTA (Kurabo Biomedical), cells were seeded in a 24-well plate in EG2 media (1,500 cells/800 µl/well). After cells reached confluence, VEGF-A (1 ng/ml) was added together with or without heparin in fresh EG2 or ocular fluid as medium. Six hrs later, HUVECs were wounded with a pipet chip by creating a “groove” in the center of well and incubated further in the same medium. The migration of the cells was stopped 24 hrs later by exchanging the medium with 4% paraformaldehyde. After cells were fixed for 20 minutes, cells were gently washed twice with PBS, and stained with 0.001% toluidine blue (Sigma-Aldrich Corp.) for 60 min. The diameter of the wound was measured from 10 areas spaced 0.71 mm from each other and averaged.

### HUVEC tube-formation assay

ECMatrix™ (Millipore Corp., Billerica, MA, USA) were applied to a 96-well plate (45 µl/well) in a cold room (4°C), which were solidified by transferring the plate in 37°C for 1 hr. After HUVECs were harvested by trypsin-EDTA (Kurabo Biomedical), cells were seeded into the matrix-coated 96-well plate in triplicate (5×10^3^ cells/150 µl/well). EGM2 media or ocular fluid was supplemented with VEGF-A (1 ng/ml) with or without heparin and used as medium. The cells were incubated for 3.0 hrs at 37°C and tube formation was stopped by exchanging the medium with 4% paraformaldehyde. After cells were fixed for 20 minutes, cells were gently washed twice with PBS, and stained with 0.001% toluidine blue (Sigma-Aldrich Corp.) for 60 min. The number of branch points and length of formed tubular structures were quantified from 4 mm×4 mm field focused in the center of the well.

### HUVEC-VEGF-165 binding assay

Fluorokine biotinylated VEGF-A (R&D Systems) was used for cell-based binding assay according to the provider's protocol. In brief, biotinylated VEGF-165 (10 µl) was added to HUVECs (1×10^5^ cells/25 µl). As a negative control, an identical sample of cells was stained with 10 µl biotinylated negative control reagent. After the samples were incubated for 60 minutes on ice,10 µl avidin-fluorescein isothiocyanate (FITC) was added to each sample, and the reaction mixture was incubated for an additional 30 minutes on ice. Then, the cells were washed twice with 1 ml of wash buffer (RDF1 buffer) and suspended in 0.25 ml of the same buffer. The stained cells were analyzed using FACS Cailbur (Becton Dickinson, San Jose, CA,USA).

### In vitro binding assays

The carboxyl terminals of heparin (10 µg/well unless designated otherwise), VEGFR1 (200 ng/well), or VEGFR2 (200 ng/well) were covalently attached to the 96-well plate coated with aminoacids (Takara Bio Inc., Shiga, Japan) at 4°C for 4 hours using ethyl-carbodiimide hydrochloride (20 mg/ml; Pierce Biotechnology, Rockford, IL, USA) as a crosslinker. After adding the blocking buffer (Takara Bio Inc.), the mixtures of VEGF-A (200 ng/ml unless designated otherwise) and heparin, HS, or the ocular fluid (100 µl/well) were applied to each well and were incubated at 4°C overnight. The plate was rinsed on ice with cold PBS and the amount of VEGF-A bound to VEGFRs was measured using components of a human VEGF ELISA kit (R&D Systems Inc.) according to the manufacturer's instructions, with following modifications. We used ice cold PBS for washing instead of washing buffer included in the kit and all the procedures were conducted at 4°C until the final washing step was completed. Finally, the plates were analyzed by measuring absorbance at 450 nm (reference at 620 nm) using a plate reader.

## Supporting Information

Figure S1 Injection of soluble VEGFR1 (sVEGFR1) terminates the development of NV in murine OIR. sVEGFR1 (0.5 µg/0.5 µl/eye; R&D systems), a potent antagonist of VEGF-A, was injected into one eye and PBS in the other at P12 in murine OIR models (N = 8). The retinal fat-mounts stained with GS lectin were subjected to analyses. Representative images of PBS-treated and sVEGFR1-treated eyes are shown (A and B, respectively). The areas of NV (red) and avascular retina (blue) relative to the entire retina (examples are shown in C and D) were quantified and expressed in percentages (E and F, respectively). NV was nearly extinguished at P17 in sVEGFR1-treated eyes (E; reduction of NV by 98.0% compared to PBS-treated eyes), while no difference was seen in the areas of avascular retina (F). All statistical data are expressed as mean ± S.E.M.
(7.85 MB TIF)
