# Supplementary figures and images for: Regulation of Pathologic Retinal Angiogenesis in Mice and Inhibition of VEGF-VEGFR2 Binding by Soluble Heparan Sulfate
Source: PLoS One. 2010 Oct 20;5(10):e13493. doi: 10.1371/journal.pone.0013493 (PMC2958111; doi:10.1371/journal.pone.0013493)

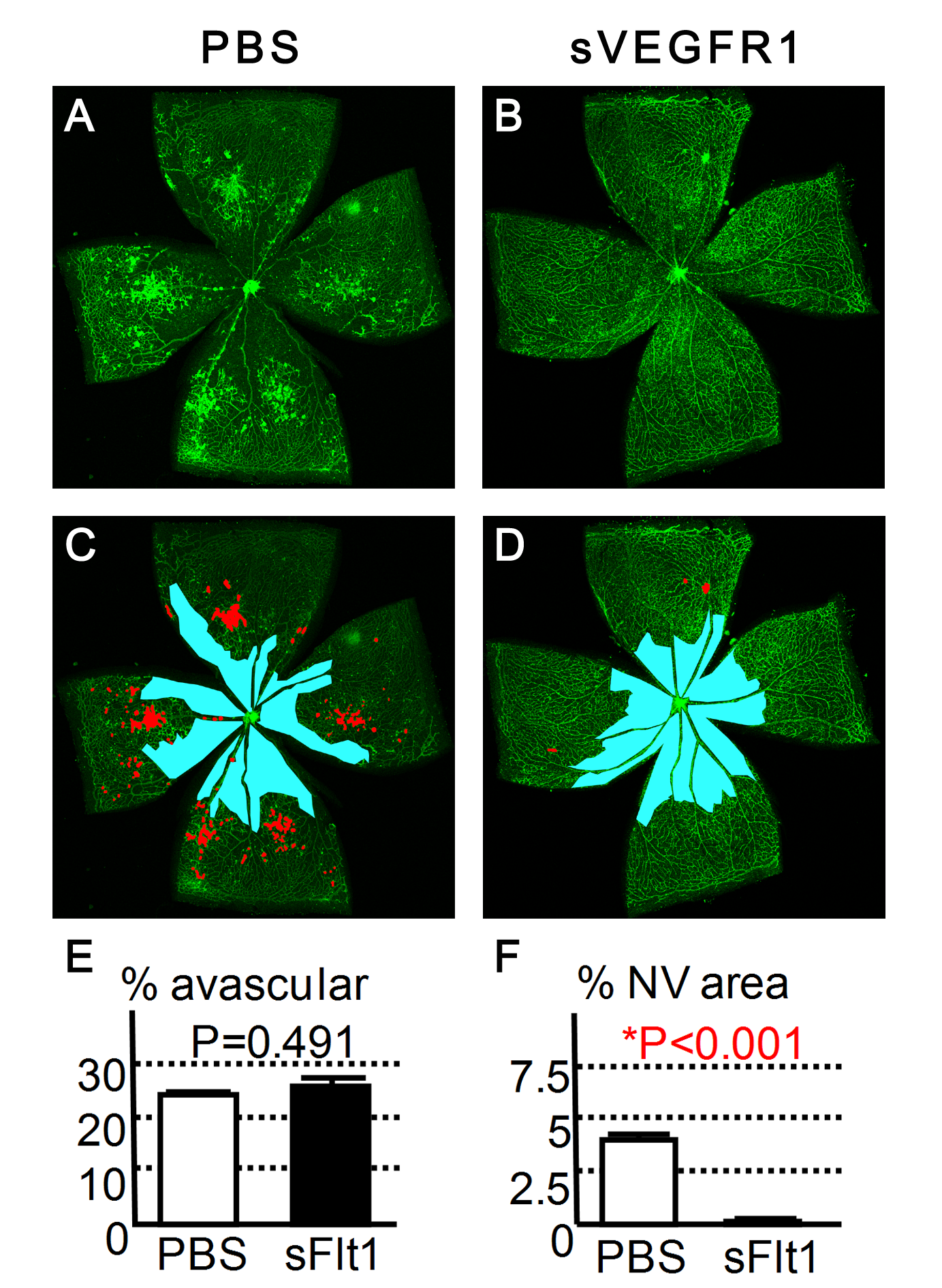

Supplement: Figure S1 — Injection of soluble VEGFR1 (sVEGFR1) terminates the development of NV in murine OIR. sVEGFR1 (0.5 µg/0.5 µl/eye; R&D systems), a potent antagonist of VEGF-A, was injected into one eye and PBS in the other at P12 in murine OIR models (N = 8). The retinal fat-mounts stained with GS lectin were subjected to analyses. Representative images of PBS-treated and sVEGFR1-treated eyes are shown (A and B, respectively). The areas of NV (red) and avascular retina (blue) relative to the entire retina (examples are shown in C and D) were quantified and expressed in percentages (E and F, respectively). NV was nearly extinguished at P17 in sVEGFR1-treated eyes (E; reduction of NV by 98.0% compared to PBS-treated eyes), while no difference was seen in the areas of avascular retina (F). All statistical data are expressed as mean ± S.E.M. (7.85 MB TIF) [file pone.0013493.s001.tif]
